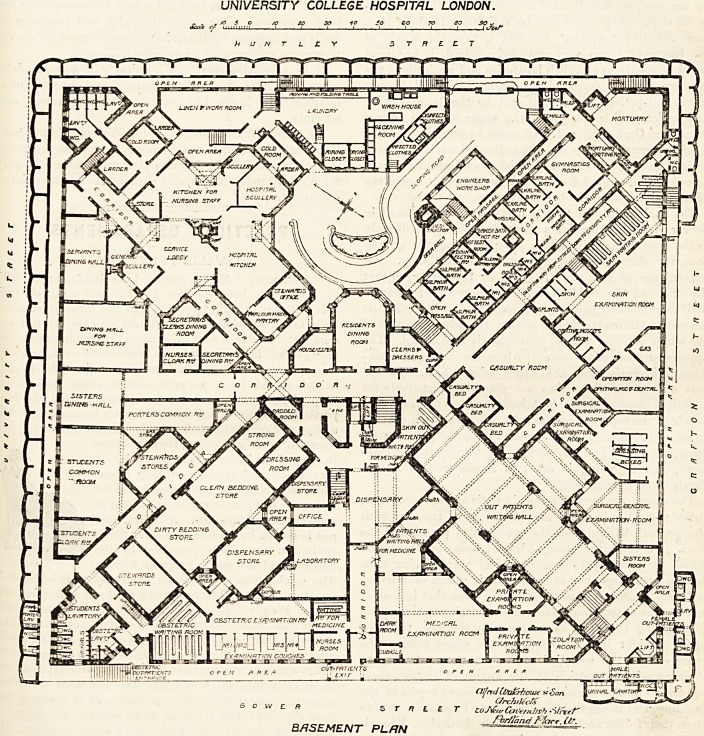# University College Hospital

**Published:** 1901-04-20

**Authors:** 


### The Institutional Workshop.

### UNIVERSITY COLLEGE HOSPITAL.

This new hospital which at present is about half completed will form a striking architectural feature at the upper end of Gower Street. It occupies a site opposite University College which includes the whole of that occupied by the old hospital as well as a considerable space at the back of it. By the acquisition of this adjoining land by the hospital authorities the site available for he purposes of the new hospital became practically a contains the staircases, the lifts, and the operating theatres. Radiating from this central block, and connected with it by short bridges, are four wings extending to each of the four angles of the site. Three of these wings contain wards, while the fourth is given up to the resident medical staff, nurses, students and servants. Each wing has at its extreme end a sanitary tower, and it is to be noticed that not only is each sanitary tower cut off from the ward or residential block to which it belongs by a "blow-through" passage of the usual hospital type, but

UNIVERSITY COLLEGE HOSPITAL LONDON.

FIRST FLOOR PLAN.

perfect square, and the generosity of Sir Blundell Maple, M.P., has provided the means for a complete reconstruction on this area.

Taking advantage of the squareness of the site the architect has laid out the building in such a way, by a diagonal arrangement of its main features, as to secure the maximum accommodation which the ground affords, and at the same time to gain for the wards the largest possible amount of light and air, while reducing the amount of space used up in corridors and means of communication to the smallest conceivable proportions. A glance at the accompanying plans will show how thoroughly this has been effected. Roughly, the buildings form a St. Andrew's Cross. The centre of the site is occupied by a block which that the ward blocks are similarly dissociated from the main block in the centre.

This arrangement was essential in a building of so many floors to prevent the passage of air from one part of the hospital to another. It is obvious enough that with a great central block, forming with its lift and staircase a vast central air shaft open from top to bottom and with the wards on every floor abutting so closely upon this air shaft as is the case in this cruciform arrangement of building, a general community of atmosphere throughout the whole building would be at once established, were it not that every ward is separated from this common staircase by a cross-ventilated bridge, in the same way as it is also separated from its own sanitary department.

As the arrangement stands this separation is fairly complete, but its maintenance in full efficiency will largely depend upon the strictness with which the discipline of the establishment is maintained. Perhaps it may be said that this is so in all hospitals, but it is clear that where the wards are separated by a certain length of corridor the chances of establishing community of atmosphere are less than where all the wards are protected from the common staircase by passages so short as these are. These will be efficient precisely in proportion as they are uncomfortable, and therefore, as we say, strict discipline will be required to maintain the "cut-off." One great advantage of the cruciform arrangement and of the connecting bridges between the central block and the wings, is that from whatever quarter the wind may blow it is caught as in a funnel in one or other of the wedge-shaped courts between the wings and is forced through the "blow-through" passages and the spaces between the bridges of connection, thereby preventing any stagnation of air in the leeward parts of the building.

The St. Andrew's Cross form of plan just described applies to the ground and upper floors, but not to the basement, where the buildings are so arranged as to cover the entire site, with the exception of a few necessary areas and the roadway leading down to the central block. Into this large basement are crowded the laundry, kitchen, and general service offices, the staff dining rooms, the students' recreation room, and the large and important out-patients' department, with its dispensary and numerous rooms for examination, dressing, &c., together with a complete series of baths. The arrangement of these rooms can best be seen by reference to the accompanying plan. A great amount of ingenuity has evidently been expended upon this problem, but it can hardly be pretended that the

UNIVERSITY COLLEGE HOSPITAL LONDON.

BASEMENT PLAN

result is entirely satisfactory. The cruciform shape of the main building, admirably as it works out upstairs, has resulted in such an assortment of strangely-shaped rooms in the basement as it would be hard to match. What portion of the linen of the establishment it is proposed to wash at home we do not know, but it strikes us that a wash-house 10 feet wide, leaving a passage hardly 5 feet wide between the tubs, is a very small affair; while as for the scullery, we find it hard to believe that the washing-up for about 300 patients can be satisfactorily done in a room not 16 feet square. The fact is that the whole of the basement seems very crowded; indeed, it has been found necessary to establish lavatories and w.c.'s under the causeways of the surrounding streets. A special feature of the out-patient department will be the large waiting hall, which will be placed in the angle between Gower Street and Grafton Street. In order to give this room a height proportionate to its other dimensions, it has been decided to abandon the ward which would naturally have occupied the ground floor of this wing, and to give the additional height to the basement room.

The main entrance to the hospital will be placed in Gower Street, from which a short corridor leads directly to the central block, in which are placed, on the ground floor, the offices of the secretary and the boardroom. For convenience of administration is has been arranged that the offices of the resident medical officer and the matron shall occupy that part of the residential wing which is nearest to the central block, so that the administration of the whole building will be conveniently centralised. The uppermost floors of the several wings are arranged somewhat differently from the stages below them. In the east and north wings they are planned respectively for isolation cases, and for diphtheria and erysipelas. The whole of the top floor of the west wing has been devoted to scientific work. At the extreme end is the post-mortem room, connected by a lift with the mortuary in the basement, and adjoining it are two laboratories, a lecture theatre, and several rooms.

The wards are very different in their arrangement from those met with in most hospitals. As is the case with the general building, so the wards also partake of the form of a cross, the straight line of beds down each side which is common to most hospital wards being thus broken by a large bay at each side, in which four or five beds are placed. It is to be noted also that instead of passing the sisters' room and various administrative offices on entering the ward, as is generally the case, the door opens straight into the ward, all the offices being at the other end in the so-called sanitary towers. Owing to their prominent position at the angles of the site these towers form the principal feature in every aspect of the building, and it has therefore been found necessary to give them their fullest architectural value in the design. Regarding them, however, from the inside it will be found that the whole of the administration of the ward is concentrated in them. For each ward there is a ward scullery, a larder, a linen closet, a nurses' w.c., a cupboard for patients' clothes (and very small it seems for the clothes of 24 patients), a bathroom, two patients' w.c.'s, and a sluice room, and in some of the wards a place is provided for a portable bath. The closets and bath are on one side of a central wall and the scullery, &c., on the other. Each wing has an external iron staircase from top to bottom of the building, running up between the wards and the sanitary towers. There is no sisters' room in connection with any of the wards, and there is no small secondary ward. The external materials used in the construction of this hospital are red brick and terra-cotta, the latter manufactured by Messrs. Doulton, and the roofs are covered with green slates. The walls of the wards have been finished internally with adamant plastering painted with oil paint. The floors are made of adamant cement on steel joists, and covered with wood, except in the staircase landings where terrazzo-mosaic has been employed. The internal angles and junctures of walls and floors and ceilings have been formed in curves to prevent any accumulation of dust.

It is calculated that the hospital will accommodate, when completed, about 300 in-patients, and the residential quarters will house 140 residents, sisters, nurses, and servants.

Mr. Alfred Waterhouse, R.A. (A. Waterhouse and Son) is the architect, and the building operations are being carried out under Mr. Thomas Holloway, acting directly for Sir J. Blundell Maple, and without the employment of a contractor.

## Figures and Tables

**Figure f1:**
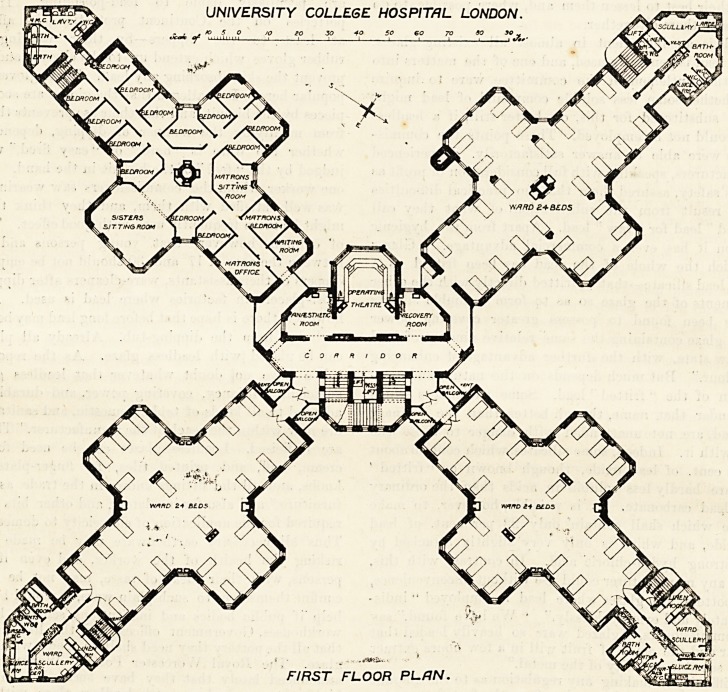


**Figure f2:**